# Time-resolved fluorescence measurements on leaves: principles and recent developments

**DOI:** 10.1007/s11120-018-0607-8

**Published:** 2018-11-26

**Authors:** Volha U. Chukhutsina, Alfred R. Holzwarth, Roberta Croce

**Affiliations:** Biophysics of Photosynthesis, Department of Physics and Astronomy, Faculty of Science, Vrije Universiteit Amsterdam and LaserLaB Amsterdam, 1081 HV Amsterdam, The Netherlands

**Keywords:** Time-resolved spectroscopy, Leaf, Re-absorption, Fluorescence

## Abstract

Photosynthesis starts when a pigment in the photosynthetic antennae absorbs a photon. The electronic excitation energy is then transferred through the network of light-harvesting pigments to special chlorophyll (Chl) molecules in the reaction centres, where electron transfer is initiated. Energy transfer and primary electron transfer processes take place on timescales ranging from femtoseconds to nanoseconds, and can be monitored in real time via time-resolved fluorescence spectroscopy. This method is widely used for measurements on unicellular photosynthetic organisms, isolated photosynthetic membranes, and individual complexes. Measurements on intact leaves remain a challenge due to their high structural heterogeneity, high scattering, and high optical density, which can lead to optical artefacts. However, detailed information on the dynamics of these early steps, and the underlying structure–function relationships, is highly informative and urgently required in order to get deeper insights into the physiological regulation mechanisms of primary photosynthesis. Here, we describe a current methodology of time-resolved fluorescence measurements on intact leaves in the picosecond to nanosecond time range. Principles of fluorescence measurements on intact leaves, possible sources of alterations of fluorescence kinetics and the ways to overcome them are addressed. We also describe how our understanding of the organisation and function of photosynthetic proteins and energy flow dynamics in intact leaves can be enriched through the application of time-resolved fluorescence spectroscopy on leaves. For that, an example of a measurement on *Zea mays* leaves is presented.

**Electronic supplementary material:**

The online version of this article (10.1007/s11120-018-0607-8) contains supplementary material, which is available to authorized users.

## Introduction

The efficient transduction of excited state energy into chemical energy constitutes the basis of photosynthesis. The timescale of this sequence of events ranges from the few femtoseconds required for exciting the pigment from a ground electronic state into an excited state, to picoseconds for collecting the excitation in the reaction centres (RCs), and picoseconds to nanoseconds to accomplish the charge separation steps in the RCs. Therefore, ultrafast time-resolved spectroscopy is the only tool to assess such processes and gain insights into the detailed reaction mechanisms. Previously, various labs have demonstrated the power of time-resolved fluorescence spectroscopy to reveal ultrafast photosynthetic dynamics in thylakoids (Vasil’ev et al. 1998; van der Weij-de Wit et al. 2007; van Oort et al. 2010), isolated photosynthetic complexes (Schatz and Holzwarth 1986; Freiberg et al. 1994; Melkozernov et al. 2004; Miloslavina et al. 2006; Jennings et al. 2013; Liu et al. 2013; Le Quiniou et al. 2015), and photosynthetic unicellular organisms such as algae (Amarnath et al. 2012; Ünlü et al. 2014; Wlodarczyk et al. 2016) and cyanobacteria (Mullineaux and Holzwarth 1991; Akimoto et al. 2012). While fluorescence induction and non-photochemical quenching (NPQ) measurements are now routinely carried out on intact leaves (Kalaji et al. 2016), performing high-quality ultrafast time-resolved measurements on such samples in the absence of artefacts is still a challenging task (Farooq et al. 2018). The first such measurements were performed by the group of Holzwarth on intact Arabidopsis leaves along with a detailed interpretation of NPQ quenching effects (Holzwarth et al. 2009; Miloslavina et al. 2011). Belgio et al. questioned some of these interpretations and proposed that measurements on isolated thylakoids and chloroplasts could be more easily interpreted and analysed (Belgio et al. 2014). However, measurements on thylakoids and chloroplasts, which should be “mimicking” the in vivo conditions, usually require addition of chemicals to keep the sample in an induced state (NPQ or state transitions) in combination with relatively short measuring times due to sample instabilities (Johnson and Ruban 2009; Wientjes et al. 2013). Some phenomena that are typical for the behaviour of intact leaves have been shown to differ substantially in isolated thylakoids/chloroplasts (Kovacs et al. 2006; Xu et al. 2015). This review describes the basic principles, important considerations and limitations, as well as the recent developments in ultrafast time-resolved spectroscopy on intact leaves.

## PAM/JTS-10 versus time-resolved spectroscopy

A common approach currently used to assess various photosynthetic parameters in intact leaves is by performing chlorophyll fluorescence induction measurements using various setups, such as Pulse-amplitude modulated (PAM) fluorimetry and a Joliot type spectrometer (e.g. JTS-10). These setups generate a signal that is proportional to the fluorescence intensity with a high signal-to-noise ratio in order to estimate the maximum quantum efficiency of PSII photochemistry, so-called “maximum PSII efficiency,” minimum (*F*_0_) and maximum (*F*_m_) Chl fluorescence levels of dark-adapted leaves (Baker 2008; Kalaji et al. 2017). The photosynthetic sample is either measured with open PSII RCs (*F*_0_ corresponding to reduced P680 and oxidised *Q*_A_, i.e. the dark-adapted state) or fully closed PSII (*F*_m_, corresponding to reduced P680 and singly reduced *Q*_A_, usually achieved by a saturating pulse of at least 2000 µE/m^2^/s of at least 150 ms duration). Maximum PSII efficiency is calculated as (*F*_m_ − *F*_o_)/*F*_m_ = *F*_V_/*F*_m_. Many other photosynthetic parameters can be estimated upon constant light illumination using the same methods. For example, PSII operating efficiency is estimated as (*F*_m_′ − *F*′)/*F*_m_′, where *F*_m_′ and *F*′ are maximal and minimal fluorescence levels measured at constant illumination (Genty et al. 1989). There are several recent reviews on these types of measurements (Murchie and Lawson 2013; Kalaji et al. 2014, 2017).

All of the interpretations of photosynthetic parameters mentioned above are greatly facilitated by the assumption that *F*_0_, *F*′, *F*_m_, and *F*_m_′ values at room temperature originate mainly from PSII. There is, however, always a contribution of PSI fluorescence present, which can be of considerable practical relevance. For example, in *A. thaliana* the PSI contribution to fluorescence is around 3% in the *F*_m_ state and 20% in the *F*_m_′ state (Tian et al. 2017). Pfündel analysed the fluorescence characteristics of 12 different species and estimated that the PSI contribution to *F*_0_ at wavelengths greater than 700 nm is about 30% and 50% in C_3_ and C_4_ plants, respectively (Pfündel 1998). The situation is made worse by the fact that essentially all commercial instruments measure these parameters by integrating the fluorescence intensity at wavelengths above 700 nm, i.e. they suppress, in order to avoid technical difficulties, the wavelength range 670–700, where PSII fluorescence is the strongest.

Secondly, under certain conditions PSI contributions might be significantly altered. For example, direct energy transfer from PSII to PSI (spillover) was proposed as an NPQ mechanism occurring in plants, algae, cyanobacteria, and lichen (Murata 1969; Chow et al. 1981; Slavov et al. 2013, 2016; Ueno et al. 2016). If present, spillover alters both the PSI and PSII contributions, so calculated photosynthetic coefficients like qI (PSII photoinhibition), qE (photoprotective deactivation of absorbed energy via the non-photochemical quenching (NPQ) process) or qT (state-transitional changes) cannot be considered to reflect only PSII effects.

Thirdly, estimation of the photosynthetic coefficients from PAM or JTS-10 type instruments cannot directly provide information regarding the actual mechanisms underlying changes in light harvesting, charge separation or its regulation. This is due to the fact that these types of measurements—even in cases where internal processes are recorded on a microsecond time scale—do not actually measure excited state fluorescence dynamics, but fluorescence intensity, i.e. the time-integrated (and in most cases wavelength-integrated) fluorescence kinetics. The relationships are given in the following way:

Usually the *fluorescence decay* signal of a complex photosynthetic system, like a leaf upon excitation with an ultrashort light pulse, can be approximated as a function *F*_f_ that depends on time *t*, excitation wavelength ${\lambda }_{\text{exc}}$ and emission wavelength ${\lambda }_{\text{em}}$ as a n-exponential decay function with n lifetimes ${\tau }_{\text{j}}$ convoluted with an instrument response function *i*(*t*).

$${F_{\text{f}}}\left( {t,{\lambda _{{\text{exc}}}},{\lambda _{{\text{em}}}}} \right)=\mathop \sum \limits_{{j=1}}^{n} {A_{\text{j}}}\left( {{\lambda _{{\text{exc}}}},{\lambda _{{\text{em}}}}} \right) \times {\text{exp}}( - t/{\tau _{\text{j}}}) \oplus i(t)$$

The lifetimes ${\tau }_{\text{j}}$ are typically in the range of femtoseconds (fs) to nanoseconds (ns). Depending on time resolution of the experimental system used, the function ${F}_{\text{f}}$ is what is measured directly in an ultrafast time-resolved fluorescence experiment. In contrast, any measurement with a PAM, JTS instrument, or any other steady-state fluorimeter measures *fluorescence intensity*, which is the time-integrated signal of the fluorescence decay, in the sense that

$${I_{\text{f}}}\left( {{\lambda _{{\text{exc}}}},{\lambda _{{\text{em}}}}} \right)=\mathop \smallint \limits_{{t=0}}^{\infty } F\left( {t,{\lambda _{{\text{exc}}}},{\lambda _{{\text{em}}}}} \right) \approx \mathop \sum \limits_{{j=1}}^{n} {A_{\text{j}}}\left( {{\lambda _{{\text{exc}}}},{\lambda _{{\text{em}}}}} \right) \times {\tau _{\text{j}}}$$

where ${I}_{\text{f}}\left({\lambda }_{\text{exc}},{\lambda }_{\text{em}}\right)$ is the wavelength-resolved *fluorescence intensity* (fluorescence emission spectrum) for a fixed excitation wavelength ${\lambda }_{\text{exc}}$, or alternatively in some cases the wavelength-resolved fluorescence intensity (fluorescence excitation spectrum) for a fixed emission wavelength ${\lambda }_{\text{em}}$. This means that most of the information on the mechanistic origin of the signals—as represented by the lifetimes and their relative amplitudes—is lost. ${I}_{\text{f}}$ is moreover not fully spectrally resolved in typical instruments (like e.g. in the PAM or JTS instruments) but the *fluorescence intensity* is integrated over a certain emission wavelength range from $\lambda _{{{\text{em}}_{1} }} \;{\text{to}}\;\lambda _{{{\text{em}}_{2} }}$

such that $$I_{f} \left( {\text{measured}} \right) = \int_{{\lambda _{{em_{1} }} }}^{{\lambda _{{em_{2} }} }} {I_{f} \left( {\lambda _{{exc}} ,\lambda _{{em}} } \right)}$$

with the disadvantage that the spectral information of the signal is lost in addition to the loss of time resolution. The great advantage of these kinds of measurements is, however, that they can be performed in a simple and straightforward manner, even with handheld instrumentation in the field (DaMatta et al. 2015). From the above formulae it follows that:

(i) All information that is contained in a steady-state fluorescence experiment (measuring *fluorescence intensity*) can be reconstructed from the time-resolved data. This also implies that as a basic principle the data from a steady-state fluorescence experiment must be consistent with the data from a time-resolved experiment, and vice versa.
(ii) Several different fluorescence decay signals—arising from different underlying mechanisms—can yield the same *fluorescence intensity* signal. Thus, a *fluorescence intensity* measurement is not capable of distinguishing between different underlying mechanisms that result in easily distinguishable *fluorescence decay* signals. One important example for photosynthetic systems is the distinction between a spillover mechanism and an antenna movement mechanism shifting antenna sizes, or absorption cross-sections. These two mechanisms can be clearly distinguished in a *time-resolved experiment*, but not in a steady-state *fluorescence intensity* measurement (Slavov et al. 2013, 2016; Chukhutsina et al. 2015).

Therefore, time-resolved fluorescence measurements allow the maximal insight into the primary steps of photosynthesis in plants in vivo while resolving details of the underlying mechanisms. Such experiments are of key importance in order to unravel energy transfer steps in different photosynthesis subunits, assess primary charge separation steps, unveil the quenching sites as well as the quenching mechanisms, and to follow the reorganisation of the photosynthetic machinery in response to environmental changes (light adaptation, NPQ, state transitions, temperature change, etc).

## Time-resolved spectroscopy on intact leaves: technical considerations

Time-resolved fluorescence measurements on intact leaves are technically challenging due to a high degree of sample complexity. Several critical issues are listed below:

- Establishment of annihilation-free conditions. Upon high excitation densities, which can occur easily in short laser pulses, multiple excited states may be created in a single photosynthetic complex, resulting in singlet–singlet (S–S) or singlet–triplet (S–T) annihilation processes between Chls. Annihilation kinetics depends not only on the excitation intensity but also on its diffusion radius, the number of pigments participating in the process, and their connectivity (Kolubayev et al. 1985; Valkunas et al. 1995). S–S and S–T annihilation processes shorten the measured fluorescence decay traces and create artefacts (van Oort et al. 2018). While annihilation kinetics can, in principle, be described by statistical approaches in isolated antenna systems e.g. (Barzda et al. 2001), for complex systems like intact leaves or organisms, the underlying theory is by far not precise enough to fully correct for annihilation effects or to separate them from internal non-annihilation kinetics, since both types of processes typically occur on the same time scale (Brüggemann and May 2003; Vengris et al. 2013; Enriquez et al. 2015). Therefore, the presence of annihilation introduces unnecessary complexity to the already complex kinetics of in vivo measurements. It is too challenging to account for such effects in data analysis. For this reason, it should be avoided at the experimental level by using carefully designed measuring conditions, such as use of proper cuvettes that allow sample refreshment, use of sufficiently low excitation intensities (see “Time-resolved fluorescence methods” and “How to achieve well-defined photosynthetic states?” sections for more details).
- PSII kinetics depends strongly on the measuring conditions: Charge separation in PSII with open RCs are much faster than with closed PSII RCs, resulting in substantially slower fluorescence kinetics in the closed state as compared to the open state. For example, isolated PSII cores in open and closed states have the reported average lifetimes of 65 ps and 850 ps, respectively (Miloslavina et al. 2006; Szczepaniak et al. 2009). Therefore, in order to be able to interpret the obtained results unambiguously, it is important to measure in a well-defined state of PSII: either at *F*_0_ (all PSII RCs open), or *F*_m_ (all PSII RCs closed) (or the corresponding *F*_0_′ or *F*_m_′ conditions). Situations with mixed states of PSII RCs yield extremely complex heterogeneous fluorescence kinetics which are then extremely difficult to analyse. A necessary requirement for the avoidance of both annihilation as well a mixed state of PSII RCs is the achievement of well-defined photosynthetic states, e.g. *F*_0_, *F*_m_, *F*_0_′, or *F*_m_′.
- A further case of RC state heterogeneity can arise in cases where very short high intensity laser pulses are used to close PSII RCs. In such situations, P680 can be oxidised instead of the usual reduced P680 state that is basically the only populated species when low intensity light sources are being used (Breton and Geacintov 1976; Geacintov et al. 1979; Geacintov and Breton 1982). Because the various RC states each have characteristic decay kinetics, heterogeneity is best avoided as the kinetic analysis becomes extremely difficult or impossible, similar to the situation where mixtures of open and closed RCs occur (see above).
- Intact leaves are susceptible to various abiotic stresses, like changes in temperature, humidity, mechanical stresses (Ahmad and Prasad 2012). Special care should be taken when handling the leaf samples to exclude the possibility of any changes during the experiment.
- Absence of spectroscopic artefacts.

Intact leaves contain fluorophores that are heterogeneously distributed over the sample depth in combination with a complex leaf morphology. Thus, two difficult situations for optical experiments are combined in a leaf: high optical density and high scattering. This can, in principle, create artefacts in the measurements that make it more difficult to extract reliable spectroscopic information: The interplay of absorption and scatter renders tissues optically turbid and distorts fluorescence (Patterson and Pogue 1994; Müller et al. 2001). However, it is primarily the spectral shape of the components which is altered, and this can be corrected (see below). Changes in the fluorescence kinetics—which would be much more disturbing for exact kinetic analysis—due to re-absorption and re-emission of fluorescence photons could, in principle, be present. They are, however, generally negligible (see below) under the typical conditions of intact photosynthetic tissues (leaves and intact algae) considering that the maximal fluorescence yields are in the range of 0.08 or less.

## Time-resolved spectroscopy on intact leaves: a historical view

The initial time-resolved fluorescence measurements on intact leaves were reported in 1980–1990s by multiple research groups (Pellegrino 1983; Schmuck and Moya 1994; Briantais et al. 1996). A huge step forward was taken by Moya and co-workers, who performed the first attempts to carry out time-resolved measurements on intact leaves in photosynthetically defined states with sufficient time resolution (10–15 ps) (Schmuck and Moya 1994; Briantais et al. 1996). The leaves were measured in *F*_s_ (steady-state fluorescence level, which is a mixed state with the contribution of both open and closed PSII) and *F*_m_ (maximum fluorescence level with reduced PSII after DCMU infiltration) states. The fluorescence kinetics observed were multi-exponential with lifetimes in the range of 25–850 ps for the *F*_s_ state and 30 ps to 2.4 ns in the *F*_m_ state, where mainly the slowest components (> 100 ps) seemed to be affected by PSII closure. The data gave the first indication that spectral separation of PSII from PSI kinetics was possible in time-resolved fluorescence measurements on intact leaves. However, since the measurement was performed with the laser excitation on the same spot of the leaf, it was impossible to achieve the *F*_o_ state (the state with minimal fluorescence intensity, corresponding to fully oxidised PSII), and S–S/S–T annihilation, which can also occur at moderate laser powers (Gruber et al. 2015), was not taken into account. More recently, fluorescence decay traces were measured on leaves during non-photochemical induction and relaxation (Sylak-Glassman et al. 2014, 2016). However, also in this case, a high contribution of S–S/S–T annihilation must also have been present during the measurements, due to measuring on the same spot of the leaf in combination with high excitation powers.

Recently, a new method of time-resolved spectroscopic measurements on intact leaves has been introduced (Holzwarth et al. 2009; Miloslavina et al. 2011; Farooq et al. 2018). It employs a rotational cuvette which is connected to a motor allowing both fast rotation as well as sideways movement (known as a Lissajous scanner). This drastically reduces the average light intensity on the leaf (up to three orders of magnitude) as compared to shining a laser at the same spot for same (usually long) total measuring time. It also eliminates physiological effects (e.g. RC state heterogeneity and S–S/S–T annihilation) caused by the measuring laser light (provided the laser repetition rate and individual pulse intensity are also optimised). This, in turn, allows the creation of specific physiological states by applying constant (high or low intensity) background light to the leaf areas which are not in the measuring beam. Thus, particular states can be produced and selected, and their fluorescence kinetics can be measured over relatively long times. Due to the low average light intensity on each leaf, the actual single pulse intensity can be much higher than in an experiment with a stationary sample, without causing artefacts or rearrangements of the photosynthetic apparatus due to high light. In addition, this method allows relatively short measuring times (in the order of 10–15 min, depending on the signal-to-noise ratio (S/N) required per wavelength decay), which is a decisive advantage since an intact leaf is a dynamic system that is difficult to maintain in the same state for an extended period of time. Finally, this kind of setup allows measurements to be performed at or very close to the native state of the plant and thus has a tremendous advantage over measurements on isolated thylakoids or chloroplasts when it comes to providing insight into in vivo processes.

Some technical considerations and limitations have to be considered when utilising a rotation cuvette for time-resolved spectroscopic measurements on intact leaves. The first one is the careful selection of the optimal diameter of the cuvette. In addition, rotation speed and side-movement of the cuvette must be optimised. In this paper, we describe a specially designed setup that allows time-resolved measurements on intact leaves. This setup is developed on the basis of the one used earlier (Holzwarth et al. 2009; Miloslavina et al. 2011). We also investigate in detail the stability of the plant leaves during the measurements and describe the possibility of measurement in defined photosynthetic states. Furthermore, the possible presence (and exclusion) of optical artefacts, which could alter fluorescence spectra and time-resolved kinetics, is addressed.

## Time-resolved fluorescence methods

Time-resolved fluorescence spectroscopy enables the observation of ultrafast dynamic events in photosynthetic systems in vivo on a ps–ns scale through direct measurement of fluorescence lifetimes. The fluorescence lifetime is a measure of the time a Chl spends in the excited state before returning to the ground state by emitting a photon. The fluorescence decay traces obtained from such measurements on leaves reflect an average over a large distribution of fluorescence lifetimes originating from Chls of different photosynthetic subunits (antenna, PSs, RCs).

Two different ultrafast kinetic techniques have been used in the recent years to follow complex fluorescence kinetics in biological samples. One is the long-established “time-correlated single photon counting technique” TCSPC. The second one is the synchroscan streak camera technique. Each has their own advantages and disadvantages. The TCSPC (or often called single-photon timing SPT) technique has the highest sensitivity and the largest dynamic range with over 4–5 orders of magnitude in both time and amplitude, which is of great benefit for analysing complex kinetics. TCSPC also allows very low excitation intensities compared to the streak camera, which are particularly crucial for maintaining distinct photosynthetic states, e.g. measuring with open PSII RCs. Disadvantages include a relatively lower time resolution compared to the streak camera technique (2–3 ps in the best instruments vs. sub-ps resolution), and the fact that, in typical instruments, each decay wavelength is measured separately. Some instrument designs are available that record the kinetics simultaneously at several wavelengths, albeit in most cases at the expense of some time resolution. For the synchroscan streak camera, however, it is typical to record the entire spectral-kinetic surface at once. The higher intrinsic time resolution of the streak camera technique comes at the expense of a substantially lower dynamic range, both in time and amplitude. In practice, for measuring intact photosynthetic tissue, the highest time resolution is not usually required (a few ps resolution is sufficient), while the complexity of the kinetics makes very high S/N ratios and very high dynamic ranges essential. Thus, in general, a well-designed TCSPC instrument has quite some advantages over the streak camera system for the measurement of complex fluorescence kinetics. However, both types of systems have been used successfully to analyse the kinetics of intact photosynthetic tissue (Freiberg et al. 1994; Melkozernov et al. 2004; Miloslavina et al. 2006, 2011; Jennings et al. 2013; Liu et al. 2013; Slavov et al. 2013, 2016; Holzwarth and Jahns 2014; Chukhutsina et al. 2015, 2017; Le Quiniou et al. 2015; Farooq et al. 2018).

We focus in the following section on the TCSPC method since it combines excellent S/N ratio, very high sensitivity, and a linear dynamic range over 4–5 orders of magnitude and it is thus our method of choice for leaf measurements.

### Time-correlated single photon counting: NIM electronics, laser sources, and detectors

Time-correlated single photon counting (TCSPC) relies on the determination of the time delay between the excitation pulse and the subsequent emission of a fluorescence photon from a sample (Fig. S1a). Single photons are repeatedly detected over many excitation cycles, and the time-dependent fluorescence signal can be reconstructed from a histogram of measured delay times (Lakowicz 1988; Karolczak et al. 2001). TCSPC electronics could be potentially comprised of independent NIM modules (see Supplementary material) or contained on a single PC board (PicoQuant GmbH 2005). NIM-based TCSPC is preferable if the user has sufficient technical skills and aims for define performance, for example the highest temporal resolution and sensitivity, since each NIM module can be chosen individually based on the required specifications. Furthermore, integrated TCSPC modules suffer from such problems like non-linearities typically raised by electronic interferences within different parts of the detection electronics (Van Hoek and Visser 1985). Such non-linearities could be avoided in NIM-based TCSPC by proper shielding of the detector and all NIM racks in combination with electric separation of different electronic elements inside the NIM rack (see Supplementary material for more details).

To obtain good reproducibility of measured fluorescence decay traces, the widths and stability of the instrument response function (IRF) are essential. The primary selection criteria for the photomultiplier tube (PMT) for an optimal IRF are a short transit-time spread of electrons and the spectral sensitivity. Due to short electron travelling distances and restricted paths, PMT microchannel plate (MCP) is the best choice according to the specifications (Hamamatsu Photonics 1994). The Hamamatsu R3809U-51 MCP PMT (our detector of choice) has the highest sensitivity in the emission region of our primary interest (600–800 nm). In combination with Ti:Sapphire excitation system it delivers an IRF width of 25–35 ps.

**Fig. 1:**
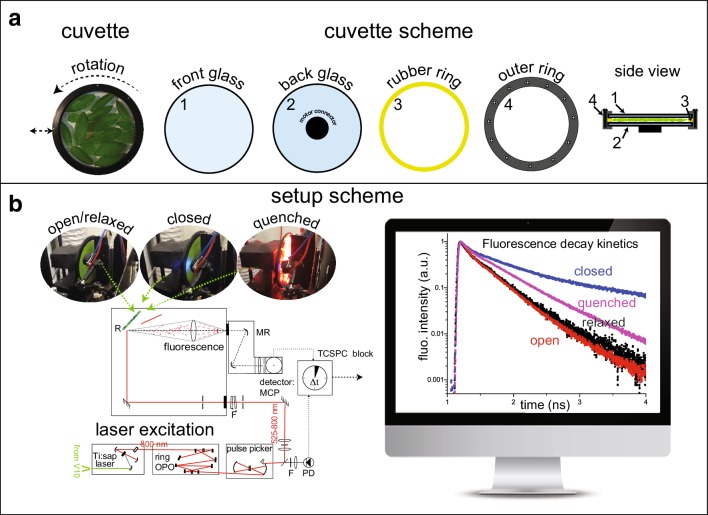
**a** Details of the rotation cuvette specially designed to measure heterogeneous, scattering samples such as intact leaves (Miloslavina et al. 2011; Holzwarth et al. 2009). It is also very useful for measuring isolated photosynthetic complexes or other liquid samples when multiple excitation effects within the same sample volume are undesirable (Miloslavina et al. 2006; Slavov et al. 2008, 2010; Szczepaniak et al. 2008, 2009). Dimensions of the cuvette: diameter 10 cm, sample thickness variable between 0.5 and 2 mm. **b** Scheme of the experimental setup that allows measurement of fluorescence decay traces on intact leaves in different physiological states: open (*F*_o_) or closed (*F*_m_) photosystem II (corresponding to minimum and maximum fluorescence levels of PSII, which occur when the electron transport carriers from PSII are in oxidised or reduced state, respectively), quenched (in the presence of NPQ, induced by illumination with strong red LEDs) and relaxed (40 min recovery in darkness after high light illumination, quenched state)

The scheme of our custom-made setup is presented on Fig. 1. The setup consists of a CW frequency-doubled Nd:YVO4 laser (Verdi V-10; Coherent) that pumps a Ti:Sapphire oscillator (Tsunami; Spectra Physics) to produce ultra-short pulses (100 fs) at 820 nm (repetition rate of ∼ 81 MHz). These pulses are fed into a synchronously pumped Ring-OPO (APE OPO), where parametric amplification and SHG (second harmonic generation) occur to deliver 200 fs pulses of a tuneable wavelength (530–720 nm). In this way, carotenoids (via green-orange light), phycobilins (via orange light), and Qy bands of Chls (via red light) can be excited. The repetition rate of the excitation is reduced by a Pulse Picker (Spectra Physics) to 4 MHz or 0.8 MHz. To resolve the fluorescence spectra of the decaying components a monochromator (DH 10 VIS, Jobin-Yvon, bandwidth about 4 nm) is employed before the PMT.

### Measuring cuvette

**Fig. 2:**
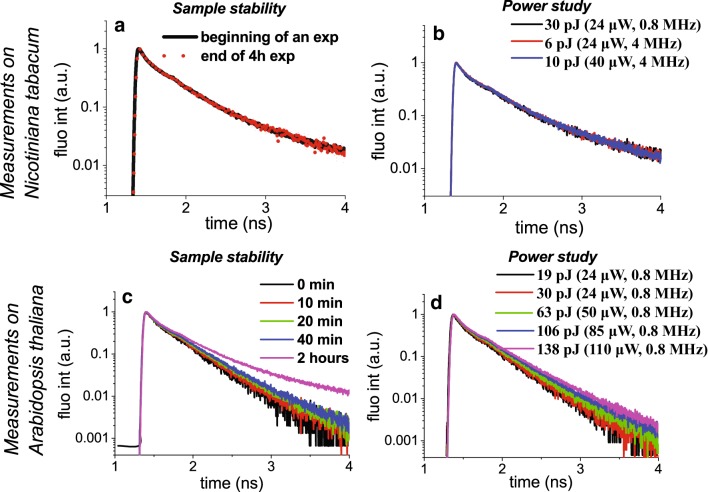
Sample stability study and power study should always be performed during time-resolved fluorescence measurements on intact leaves and isolated samples. The checks are performed at a characteristic wavelength, 686 nm, which is close to PSII and free Chl emission maxima. **a, b** Measurements on *Nicotiana tabacum* leaves in *F*_o_ state. Rotation speed was 1200 rpm. Side movement was 80 mpm. **a** Sample stability was confirmed by measuring fluorescence decay traces at the beginning and end of the experiment. **b** The power study demonstrated that the fluorescence kinetics are not affected in the range between 20 and 60 µW (corresponding to the pulse energy of 6–30 pJ). **c, d***Arabidopsis thaliana* leaves require different measuring conditions than *N. tabacum* to achieve *F*_o_ state. **c** Rotation speed was 1200 rpm. Side movement was 80 mpm. Sample stability was tested by measuring fluorescence decay traces every 10 min during the experiment. The stability study demonstrated that *A. thaliana* leaves cannot withstand the same rpm as *N. tabacum*: irreversible damage to the leaves occurs after only 40 min of rotation. Experimentally, a slower speed of 700 rpm was determined to be optimal for *A. thaliana* leaves. **d** Power study of *A. thaliana* leaves upon 700 rpm rotation: no more than 25 µE should be used to achieve *F*_o_ state at speeds of 700 rpm

**Fig. 3:**
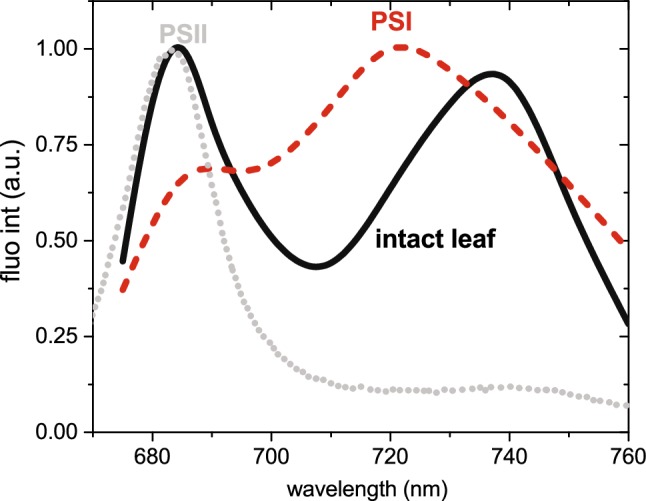
Steady-state emission spectrum obtained from an intact leaf. The fluorescence was dominated by two emission bands: 682 nm, characteristic for PSII (dotted line) and Light-harvesting complex II, and 720–740 nm, characteristic for PSI emission (dashed line)

To study photosynthetic response of intact leaves, the majority of fluorescence measurements are made from the upper side of the leaves (adaxial, i.e. facing the sun), where the primary photosynthetic tissue, the palisade mesophyll, is located. Due to high scattering and re-absorption, the time-resolved measurements are performed only in a front-face configuration, i.e. measurement from the same side as excitation. For such measurements a custom-made cuvette was used. The cuvette, composed of two glass plates with a rubber ring in between, is tightly closed with two metal plates (Fig. 1a). This tight enclosure allows the immersion of detached plant leaves in a buffered sucrose solution (0.3 M) during the measurements in order to maintain the osmotic pressure of the leaf tissues and prevent the loss of cellular fluids due to centrifugal force (Fig. 2, diameter = 10 cm, thickness 1–2 mm, Fig. 3a). Typical rotation speeds of the cuvette used for these experiments were 600–1200 rpm (depending on the plant species). The cuvette can also oscillate laterally with a typical speed of 76 side movements per minute. This movement ensures a very low average laser intensity per sample area such that potential secondary effects induced by the laser can be excluded (see “Time-resolved spectroscopy on intact leaves: technical considerations”). The use of only low excitation powers combined with the rotation and lateral motion of the cuvette allows fluorescence detection of the photosynthetic apparatus in any possible physiologically relevant state, while maintaining that state over a longer period of time (in the order of up to hours) (see Fig. 1b).

## How to achieve well-defined photosynthetic states?

The measurements on the setup described above can be performed on four well-defined photosynthetic states (Fig. 1b): open (*F*_o_), closed (*F*_m_), fully quenched (*F*_m_*′*) and recovered (*F*_rec_).

1. *F*_o_ and *F*_rec_: *F*_o_ (open state) is measured in complete darkness after overnight dark-adaptation. Pulse energy for the detection of F_o_ should be typically in the range of 25–75 pJ and the rotational speed should be in the range of 700–1200 rpm. It is crucial to perform preliminary checks with different laser powers, repetition rates and rotation values to ensure that the PSII RCs are indeed open and there is no damage of the leaves due to the rotation. The measurements in the recovered state are done with the same measurement settings as for *F*_o_ state. *F*_rec_ is typically reached after 1 h of recovery in darkness after *F*_m_′ measurement.
2. *F*_m_: To measure on leaves with closed PSII reaction centres (closed state), the dark-adapted leaves should be incubated in DCMU solution: Our standard procedure included 6–12 h of incubation of intact leaves in sucrose (0.3 M) and 50 µM DCMU at 4 °C in darkness (the time of incubation depended on the plant species and leaf sizes). After the incubation, *F*_v_/*F*_m_ was measured in the leaves using FluorCam (Photon Systems Instruments) and only the leaves with even *F*_v_/*F*_m_ distribution below 0.2 were used. During time-resolved experiment, to ensure that all PSII RCs are indeed closed, an additional weak blue LED (~ 50 µmol photons m^−2^ s^−1^) is focused in a 1-cm-diameter spot onto the rotational cuvette right above the site of excitation by the laser light pulses (Fig. 1b) (1 mm diameter). To check whether the full closure of PSII is achieved, different intensities of blue light should be tested. In the end, the blue led intensity should be set to the minimum value that permits reaching the maximum fluorescence yield.
3. Quenched state *F*_m_*′*. Light-adaptation for the *F*_m_*′* state is carried out using a mixed array plate of red high-intensity light-emitting diodes allowing up to 1000 µmol photons m^−2^ s^−1^ actinic light. Measurements should be started only when the level of NPQ is stabilised (depending on the growth conditions and the species, it typically requires 30–40 min of illumination). To prevent CO_2_ limitation during illumination, additional carbon sources (50 mM NaHCO_3_) are added to the buffer. Additionally, for closing PSII RCs under quenched conditions, a blue high intensity LED is focused in a 1-cm-diameter spot onto the rotational cuvette right above the fluorescence excitation laser light pulses (of 1 mm diameter). This allows full closure of the PSII RCs. The arrangement and condition must be checked carefully by comparison ${\text{NPQ}}\left( {=\frac{{{\tau _{{\text{avg}}}}\left( {{F_{\text{m}}}^{\prime }} \right)}}{{{\tau _{{\text{avg}}}}\left( {{F_{\text{m}}}} \right)}} - 1} \right)$ value obtained from the time-resolved experiment with the one obtained by fluorescence induction techniques.

### Power and stability study

Numerous quality control measures must be employed to ensure the photosynthetic machinery is in a defined photosynthetic state. The necessary checks are presented in Fig. 2. The measurements shown in Fig. 2a, b were performed on a tobacco leaf in *F*_o_ state. It is always crucial to check sample stability, since leaf tissue is susceptible to mechanical stresses and senescent processes (Biddington 1986). Cuvette rotation that is too fast or an extended measurement period can damage the leaves and alter the fluorescence kinetics. Figure 2a shows the fluorescence decay traces at a characteristic wavelength at the beginning and end of a 4-h experiment on *N. tabacum*. The two fluorescence decay traces are indistinguishable, confirming sample stability within the experimental timeframe. In agreement, the *F*_v_/*F*_m_ value (which is a widely used measure of plant fitness) of the same leaf before and after the measurement did not change (0.81). The laser power-dependency study (Fig. 2b) is required to confirm that the measurements are indeed performed in *F*_o_ state. This control also excludes the presence of any laser-related artefacts. Therefore, the detection wavelength used in these control measurements should be close to that of free Chl (to detect leaf damage) or PSII (to detect PSII closure) emission maxima. Here, *λ*_det_ = 686 nm was used. To achieve *F*_o_ state in tobacco leaves, the combination of a rotational speed of 1200 rpm and pulse energy of 6–10 pJ (4 MHz)/25 pJ (0.8 MHz) was used. Considering the diameter of the laser beam (1 mm) and the cuvette dimensions (Fig. 1), the laser beam takes ∼ 160 µs to pass through the sample volume before the dark interval (0.8 s). This means that before the dark interval, the sample volume is hit by 130 pulses (if the set repetition rate is 0.8 MHz) or by 640 pulses (if the set repetition rate is 4 MHz). As a result, the summed energy of the excitation pulses applied to *N. tabacum* is 3–6 µJ.

Experiments on *A. thaliana* performed with the same rotational speed used for *N. tabacum* (1200 rpm) demonstrated a severe disturbance of the photosynthetic machinery after only 40 min, as evidenced by a longer-lived fluorescence decay trace (Fig. 2c). In agreement with this observation, the *F*_v_/*F*_m_ of the *A. thaliana* leaves dropped to 0.4 after 2 h of rotation at 1200 rpm. For *A. thaliana* is thus necessary to use a slower rotation speed. Numerous experiments with different rotational speeds led to the conclusion that 700 rpm did not cause any damage to *A. thaliana* leaves within the experimental timeframe (4–6 h). However, slower rotation of *A. thaliana* as compared to *N. tabacum* led to 1.7 times higher average power per sample area. In agreement, the laser power dependency study demonstrated that to achieve *F*_o_ state in *A. thaliana*, the pulse energy needed to remain below 30 pJ. With a laser repetition rate of 0.8 MHz resulting in 218 pulses, the maximal total energy a sample volume of *A. thaliana* received before the dark period was 6.5 µJ, which was very similar to the value observed for *N. tabacum* leaves (Fig. 2d).

### Checks for the presence of optical artefacts

Fluorescence spectra of intact leaves are characterised by two bands: one in the red region of the visible spectrum (at about 685 nm, F685) and another in the near infrared region (at about 735 nm, F735) (Fig. 3). As the overlap between absorption and emission spectra is higher in the red spectral region than in the NIR, the 685-nm band is the most affected by re-absorption.

It is known that re-absorption alone only affects the steady-state fluorescence spectra but not the fluorescence kinetics (Lakowicz 1994; Terjung 1998). However, in heterogeneous and highly scattering samples like animal tissues, minerals or polymer blends, re-absorption can be combined with other optical effects, like re-emission and time-of-flight dispersion (dispersion of the incident and the emitted photons in spatially extended samples), both of which modulate fluorescence decay traces (Lakowicz 1994). It was demonstrated that the presence of re-emission slows down fluorescence kinetics (Mammel et al. 1992; Connolly et al. 1982; Lakowicz 1994). The impact of re-emission on measured fluorescence lifetimes can be quite substantial in a solution of highly concentrated Chls (Connolly et al. 1982), which was reported to have fluorescence yield of around 0.33 (Brody 2002). However this effect can be avoided or minimised if very thin layers of photosynthetic tissue are used (Lakowicz 1994) or if the measurements are performed in front-face detection (Connolly et al. 1982). Also, in intact leaves, up to 1–6% of the absorbed light is released as fluorescence depending on the state of photosynthetic machinery, suggesting that the effect of re-emission should be negligible.

**Fig. 4:**
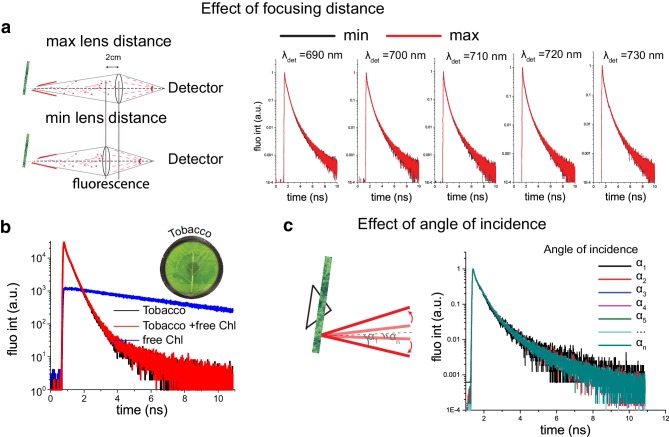
**a, b** Test of the presence of re-emission in time-resolved decay traces of intact leaves. **a** The decay traces were measured at various detection wavelengths in two extreme positions of the focusing lens: (1) the surface of the leaf; (2) into the leaf tissue. **b** Time-resolved fluorescence traces obtained from: (1) *N. tabacum* leaf (black line), (2) free Chl (blue line), and (3) the same *N. tabacum* leaf together with a Chl filter paper, when the latter is positioned on the back side of the cuvette (red line). Fluorescence kinetics measured from intact leaves (with/without Chl in the back) are indistinguishable. **c** The presence of time-of-flight dispersion is tested: time-resolved decay traces were measured at different angles of incidence. The angle of incidence does not affect fluorescence kinetics, excluding the possible contribution of time-of-flight dispersion

To confirm the absence of re-emission in our data, we checked how changes in the focus depth affect the fluorescence kinetics. We measured fluorescence kinetics at two utmost positions of the focusing lens: with the focus (1) on the surface of the leaf; and (2) into the leaf tissue (Fig. 4a). The resulting fluorescence kinetics were undistinguishable, confirming absence of re-emission in our fluorescence decay traces. This result suggests that independently on the focus position we always measure fluorescence kinetics from a thin layer of cells on the surface of the leaf and thus that re-emission does not contribute to the measurements. To confirm this conclusion, a filter paper covered with isolated chlorophylls was placed into the rotational cuvette. Measured fluorescence decay trace from the filter paper with Chls alone showed long-lived decay traces as expected (Fig. 4b). Next, we measured the time-resolved fluorescence traces from a tobacco leaf alone and from the leaf together with the filter paper with Chls, with the latter positioned on the back side of the cuvette. The fluorescence kinetics measured on leaves with and without the posterior Chls were indistinguishable (Fig. 4b), supporting the validity of measurements obtained from the surface of the leaf.

To exclude the possibility that time-of-flight dispersion (due to scattering inside the leaf tissue) affects fluorescence decay traces, we also studied whether changes in the angle of incidence affect the fluorescence kinetics. Figure 4c shows that the fluorescence decay traces upon excitation at different angles of incidence were indistinguishable. Therefore, we can confirm that the fluorescence kinetics measured in a front-face configuration were not affected by the heterogeneous structure of the leaf tissue.

## Example: measurements on *Zea mays* in *F*_m_ state

### Global analysis

**Fig. 5:**
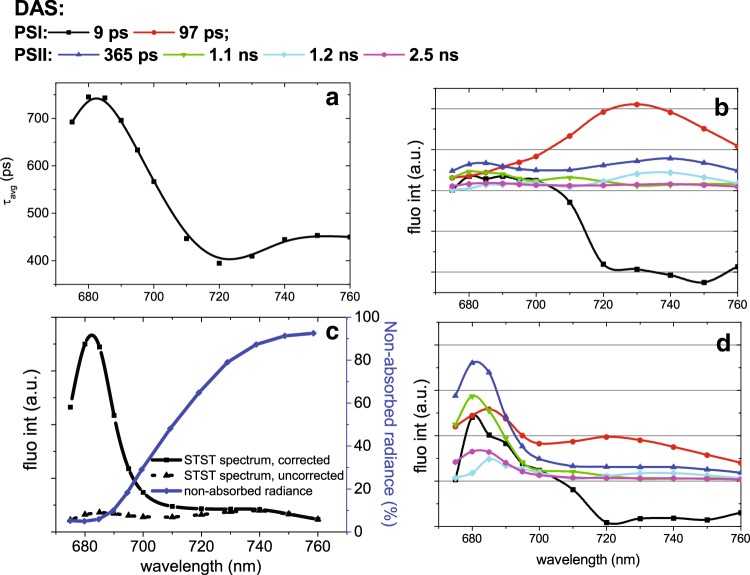
**a** Average lifetime of *Z. mays* leaves in *F*_m_ state at different detection wavelengths; **b, d** DAS obtained from a 6-component fit of fluorescence decay traces. Original DAS (**b**) and the DAS corrected for re-absorption (**d**). **c** Correction of Chl re-absorption in fluorescence measurements on intact leaves. Spectra of the measured chlorophyll fluorescence emission (black dotted line, *F*_m_) are corrected for re-absorption according to Formula 1 of the main text. The radiation not absorbed by *Z. mays* leaves (transmitted and reflected light, parameter *r* + *t* in Formula 1) is indicated with a blue line

Time-resolved fluorescence experiments were performed on *Z. mays*, one of the keystone model organisms for photosynthesis research. Maize leaves were measured in *F*_m_ state. The plants were grown under normal light conditions (120 µE) for 10 days and dark-adapted before the experiments. To resolve spectra of different emitting species, we measured fluorescence decay traces at eight detection wavelengths between 670 and 760 nm, since this was shown to be sufficient to resolve the kinetic processes and spectra of an intact Arabidopsis leaf in detail (Holzwarth et al. 2009; Miloslavina et al. 2009). In Fig. 5 we present results obtained from the global analysis [method as described previously (Holzwarth 1995, 1996; van Stokkum et al. 2004)]. In short, the measured fluorescence decay traces were analysed globally with a number of parallel, non-interacting decays. So, the experimental kinetics function *f* (*t, λ*) was fitted as:

$$f\left( {t,\lambda } \right)=\mathop \sum \limits_{{i=1,2}}^{N} DA{S_{\text{i}}}(\lambda ){\text{exp}}\left( { - \frac{t}{{{\tau _i}}}} \right) \oplus i(t),$$

where *DAS*_i_ (Decay-Associated Spectra) is the amplitude factor associated with decay component *i* having decay lifetime *τ*_i_, and *i*(*t*) is the instrument response function. The symbol $\oplus$ denotes time convolution of the two functions. Six DAS were needed to fit the decay traces satisfactory at all detection wavelengths (Fig. 5). The average lifetime decreased from 700 to 400 ps going from the red region (670–700) to the NIR (700–760) (Fig. 5a). This was expected since relatively short-lived fluorescence from PSI emits predominantly in the NIR region (Croce et al. 2000; Wientjes et al. 2011a). The two fastest components (9 ps and 97 ps) (Fig. 5b) have lifetimes and spectra typical of PSI (Croce et al. 2000; Wientjes et al. 2011a; Jennings et al. 2013; Tian et al. 2017). The remaining DAS (365 ps, 1.1 ns, 1.2 ns, 2.5 ns) (Fig. 5b) were putatively assigned to PSII kinetics: Their spectra had substantial emission in the 685-nm region, corresponding to the PSII maximum. The lifetimes are also close to the ones reported for closed PSII (Szczepaniak et al. 2009). The total average lifetime for PSII-related components was ∼ 0.9 ns.

**Fig. 6:**
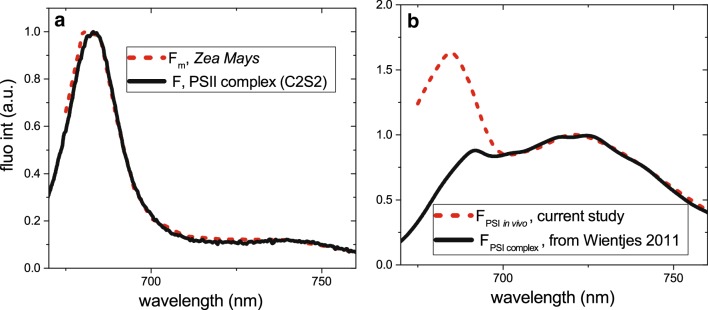
**a** Reconstructed steady-state emission spectra obtained from time-resolved measurements on intact leaves of *Z. mays* in *F*_m_ state. The spectra are corrected for re-absorption. The resulting spectra were compared to that of isolated Photosystem II [C_2_S_2_ from (Xu et al. 2015)]. **b** Spectrum of PSI in vivo compared to the spectrum of the isolated PSI [from (Wientjes et al. 2011b)]

Due to the high concentration of photosynthetic pigments inside the leaf tissue, the measured fluorescence spectra are strongly affected by re-absorption. Although re-absorption does not influence the fluorescence decay kinetics, it affects the intensity measured at different detection wavelengths, and, as a result, it changes the shape of the emission spectra of the time-resolved components (Fig. 5b). All the components have a reduced emission in the 670–700 region due to re-absorption. For example, PSI emission in vitro has comparable amplitudes in the 685-nm and in the 720-nm regions (Fig. 6) (Wientjes et al. 2011b), while PSI-related components measured on intact leaves hardly have any 685-nm signatures (Fig. 5b). Re-absorption strongly affects the PSII-related spectra. The vibration band emitting around 720 nm was nearly the same amplitude as the 680 nm emission (Fig. 5b), while in re-absorption–free conditions the amplitude of the vibration band was reduced by 10–20% with respect to the 680 nm peak (Andrizhiyevskaya et al. 2005; Xu et al. 2015) (Fig. 6).

### Correction of steady-state and time-resolved spectra from re-absorption effects

Correction of the time-resolved spectra for re-absorption would allow a better interpretation of the data. Various models have been suggested to account for re-absorption in the emission spectra (Mammel et al. 1992; Müller et al. 2001). Experimentally, Gitelson et al. (1998) demonstrated that the real F685/F735 ratio of steady-state fluorescence in intact leaves is closely related to the ratio of non-absorbed light at 685 nm and 735 nm. Other reports (Agati et al. 1993; Lagorio et al. 1998; Ramos and Lagorio 2004) used defined physical approaches to describe the interaction between light and matter to quantify re-absorption more precisely. Both Agati et al. and Lagorno et al. models were demonstrated to be well-suited to reliably account for re-absorption. The model of Agati and colleagues was suggested to be preferable in case of measurements on non-overlapping leaves (Cordon and Lagorio 2006), and was therefore used for correcting the spectra in Fig. 5b.

In short, fluorescence spectra (*F*(*λ*)) were corrected for light re-absorption (*Fcorr* (*λ*)) using the following formula:

$$Fcorr \left(\lambda \right)=F\left(\lambda \right)\times f$$, where

1 $$f=\frac{{ln\frac{1}{{({r_{{\lambda _0}}}+{t_{{\lambda _0}}})}}+ln\frac{1}{{({r_\lambda }+{t_\lambda })}}}}{{n\frac{1}{{({r_{{\lambda _0}}}+{t_{{\lambda _0}}})}}}} \times \frac{{1 - {r_{{\lambda _0}}} - {t_{{\lambda _0}}}}}{{1 - ({r_{{\lambda _0}}}+{t_{{\lambda _0}}})({r_{{\lambda _0}}}+{t_{{\lambda _0}}})}}$$

In this formula, *r* and *t* stand for reflected and transmitted radiation, respectively, while *λ*_0_ and *λ* are the excitation and detection wavelength, respectively (Fig. 5c). *R* and *t* spectra were recorded as a function of wavelength for individual leaves using a spectrophotometer (Varian Cary 4000 UV–Visible spectrophotometer) equipped with an integrating sphere. The same leaves used for *r* and *t* measurements were used in the time-resolved fluorescence experiments from Fig. 5.

Figure 5d shows the DAS after correction for re-absorption. PSI- and PSII-related components now can be visually distinguished. The reconstructed steady-state spectrum of *F*_m_ state is presented in Fig. 6. As expected, the reconstructed steady-state *F*_m_ spectrum does look like the spectrum of isolated PSII, confirming the validity of the approach (Fig. 6a). The reconstructed steady-state *F*_m_ spectrum is dominated by PSII emission (95%), while PSI emission contributes only to 5% of its area. After spectra correction, it becomes clear that PSI, decaying within 97 ps, is dominated by 685-nm emission, unlike that observed in isolated PSI–LHCI complexes (Fig. 6b). This suggests that there is an additional LHCII attached to PSI–LHCI complexes in darkness in vivo in maize, which is unexpected since in dark-adapted leaves LHCII are usually considered to be associated only with PSII.

## Summary

In this work, we have described the methodology of time-resolved spectroscopy on intact leaves. The combination of highly sensitive detection, high time resolution and usage of a rotation cuvette allowed measurements on intact leaves in physiologically relevant photosynthetic states: open (PSII oxidised), closed (PSII reduced), quenched (upon high-light illumination) and relaxed (after recovery from light stress). Using the described methodology, any possible optic artefacts affecting fluorescence decay traces were avoided. Furthermore, decay-associated spectra as well as steady-state emission spectra can be corrected for the re-absorption effect allowing a straightforward interpretation. Measurements on intact leaves allowed the assessment of photosynthetic dynamics on the ps timescale in vivo. An example of time-resolved measurements on maize leaves was described, which yielded new insights of PSI dynamics in vivo.

## Electronic supplementary material

Below is the link to the electronic supplementary material.

Supplementary material 1 (DOCX 57 KB)
