# Supplementary material for: Time-resolved fluorescence measurements on leaves: principles and recent developments
Source: Photosynth Res. 2018 Nov 26;140(3):355–69. doi: 10.1007/s11120-018-0607-8 (PMC6509100; doi:10.1007/s11120-018-0607-8)
Supplement: Supplementary file 1 — Supplementary material 1 (DOCX 57 KB) [file 11120_2018_607_MOESM1_ESM.docx]

**Supporting material**

**Time-resolved fluorescence measurements on leaves: principles and recent developments”**

Volha U. Chukhutsina, Alfred R. Holzwarth, Roberta Croce

**NIM-based TCPSC**

Time-correlated single photon counting (TCSPC) relies on the determination of the time delay between the excitation pulse and the subsequent emission of a fluorescence photon from a sample (Fig. S1a). Single photons are repeatedly detected over many excitation cycles, and the time-dependent fluorescence signal can be reconstructed from a histogram of measured delay times (Lakowicz 1988; Karolczak et al. 2001). The TCSPC setup used in this work is implemented in a “reverse” mode (Fig. S1b). This means that not the time between an excitation pulse and detection of a resulting photon is measured, but rather the time between the detection of a photon and the electronically delayed excitation laser pulse. After a short (<0.2 ps) laser light pulse excites the sample, emission of an individual photon is detected by a microchannel plate photomultiplier tube (MCP-PMT, HAMAMATSU R3809U-51) and amplified by an amplifier (Hewlett Packard 8447F). The signal is split: one part goes to a constant fraction discriminator (CFD, TC 454 TENNELEC), one is directed to a differential discriminator (DD). The differential discriminator (EG&G TD 101/N) selects only those pulses whose voltage amplitudes lie within a set window between given minimum and maximum voltage values. It is connected to TAC via STROBE function, which allows an external control signal to determine if a TAC output is generated. CFD, once receives a single photon pulse from the MCP-PMT, generates an electronic pulse, which successively starts a time-to-amplitude converter (TAC, TC 862 TENNELEC) if the strobe signal is received from DD. The excitation pulses are fed via a photodiode into another CFD (TC 454 TENNELEC), which sends a stop signal to the TAC upon arrival of the pulse. To allow the excitation pulse to be used as a stop signal, its arrival to the CFD has to be electronically delayed respect to start pulse. This is typically achieved through a suitable fixed time delay provided by a 50 Ω delay line. After a “start” signal due to the detection of a photon, the “stop” signal will therefore arrive with a fixed delay after the original excitation pulse.

After receiving a start signal, the TAC develops a voltage that increases linearly in time. After the stop signal, the TAC voltage is converted to a numerical value by an analog-to-digital converter (ADC 7423 UHS Selena Milano). The output of the ADC is gathered in 8192 channels of a custom-made analyser, which sorts the voltage values according to counts at each voltage (≅ time).

Detector and electronics have a finite recovery time before they are able to detect the next photon. When another fluorescence photon appears, after the TAC has been started, the timing information of that second photon will be wasted. That will lead to “pile-up distortion”. To avoid pile-up distortion the probability to detect more than one photon per excitation pulse should be negligible. As a rule of thumb the photon counting rate should not be more than 1 photon per 100-200 laser pulses. This criterion is typically achieved by reducing the energy of the excitation pulses with neutral density filters to the pJ level. Under operating conditions the rate of detected fluorescence photons are typically set to about 20,000 per second (<1% of the number of excitation pulses per second) if the repetition rate of a laser is 4 MHz or 8000 if it is 0.8 MHz. For instrument response (IRF) measurements it is a good practice to use the excitation powers, which would lead to around one photon in 500-1000 excitation pulses. In this way a better resolution of a small long tail due to delayed electrons, which is always present in all MCP-PMTs’ IRFs, can be achieved. Sometimes electromagnetic interference signals can arise in addition to the start and stop signals. The signals can couple inside the NIM electronics for example between the two CFDs or between CFD and differential discriminator. There may also be a spurious pickup of some other start- or stop-related signals: routing signals, switching signals from pulse pickers, or signals from the driver of a pulsed laser diode. Any signal that is synchronous with the recorded light signal is a potential source of increased differential nonlinearity, which decreases time-resolution of the setup as a result. The most efficient way to avoid such problems is proper shielding of the detector and all NIM racks in combination with electric separation of different electronic elements inside the NIM rack and a precisely designed principle to provide electronic grounding only at one point (essentially at the end point of the signals).

Supplementary Figure 1 Principles of time-resolved fluorescence measurement with TCSPC. **a** The histogram of photon arrivals per each time delay is constructed and yields a fluorescence decay curve. **b** The time delay τ between the excitation pulse and the single photon emitted by the fluorescing sample is converted by a time-to-amplitude converter (TAC) to an output voltage signal. Then the Analog-to-Digital Converter (ADC) is used to resolve the signal from the TAC into thousands of time channels and write into the corresponding address of the memory. For the details see the text.

References:

Karolczak J, Komar D, Kubicki J, Wróżowa T, Dobek K, Ciesielska B, Maciejewski A (2001) The measurements of picosecond fluorescence lifetimes with high accuracy and subpicosecond precision. Chemical physics letters 344 (1):154-164

Lakowicz JR (1988) Principles of frequency-domain fluorescence spectroscopy and applications to cell membranes. Sub-cellular biochemistry 13:89-126
